# SmMIP-tools: a computational toolset for processing and analysis of single-molecule molecular inversion probes-derived data

**DOI:** 10.1093/bioinformatics/btac081

**Published:** 2022-02-12

**Authors:** Jessie J F Medeiros, Jose-Mario Capo-Chichi, Liran I Shlush, John E Dick, Andrea Arruda, Mark D Minden, Sagi Abelson

**Affiliations:** Princess Margaret Cancer Centre, University Health Network (UHN), Toronto, ON, Canada; Ontario Institute for Cancer Research, Toronto, ON, Canada; Department of Molecular Genetics, University of Toronto, Toronto, ON, Canada; Genome Diagnostics, Department of Clinical Laboratory Genetics, University Health Network, Toronto, ON, Canada; Department of Immunology, Weizmann Institute of Science, Rehovot, Israel; Princess Margaret Cancer Centre, University Health Network (UHN), Toronto, ON, Canada; Department of Molecular Genetics, University of Toronto, Toronto, ON, Canada; Princess Margaret Cancer Centre, University Health Network (UHN), Toronto, ON, Canada; Princess Margaret Cancer Centre, University Health Network (UHN), Toronto, ON, Canada; Department of Hematology and Medical Oncology, University Health Network, Toronto, ON, Canada; Ontario Institute for Cancer Research, Toronto, ON, Canada; Department of Molecular Genetics, University of Toronto, Toronto, ON, Canada

## Abstract

**Motivation:**

Single-molecule molecular inversion probes (smMIPs) provide an exceptionally cost-effective and modular approach for routine or large-cohort next-generation sequencing. However, processing the derived raw data to generate highly accurate variants calls remains challenging.

**Results:**

We introduce SmMIP-tools, a comprehensive computational method that promotes the detection of single nucleotide variants and short insertions and deletions from smMIP-based sequencing. Our approach delivered near-perfect performance when benchmarked against a set of known mutations in controlled experiments involving DNA dilutions and outperformed other commonly used computational methods for mutation detection. Comparison against clinically approved diagnostic testing of leukaemia patients demonstrated the ability to detect both previously reported variants and a set of pathogenic mutations that did not pass detection by clinical testing. Collectively, our results indicate that increased performance can be achieved when tailoring data processing and analysis to its related technology. The feasibility of using our method in research and clinical settings to benefit from low-cost smMIP technology is demonstrated.

**Availability and implementation:**

The source code for SmMIP-tools, its manual and additional scripts aimed to foster large-scale data processing and analysis are all available on github (https://github.com/abelson-lab/smMIP-tools). Raw sequencing data generated in this study have been submitted to the European Genome-Phenome Archive (EGA; https://ega-archive.org) and can be accessed under accession number EGAS00001005359.

**Supplementary information:**

Supplementary data are available at *Bioinformatics* online.

## 1 Introduction

Sensitive and cost-effective targeted next-generation sequencing (NGS) can enable a myriad of clinical applications, including screening of microbial populations (Deurenberg *et al.*, 2017), non-invasive prenatal testing (Gregg *et al.*, 2014), and early cancer detection (Karlovich and Williams, 2019). It can help evaluate drug efficacy in clinical trials, improve routine diagnostic testing of tumours in molecular diagnostic laboratories and inform cancer treatment decisions by longitudinal sequencing efforts to monitor emerging treatment-resistant clones (Karlovich and Williams, 2019). Despite this immense potential, many NGS approaches being used for research and clinical applications remain time-consuming, costly, and incredibly difficult to scale.

Single-molecule molecular inversion probes (smMIPs) provide a highly practical, cost-effective approach for multiplex-targeted genomic capture (Mamanova *et al.*, 2010). When compared with other targeted sequencing techniques, these single-stranded oligos simplify the creation of NGS libraries towards the discovery of genetic variations (Porreca *et al.*, 2007; Turner *et al.*, 2009). Molecular inversion probes have undergone a series of advances over the years allowing researchers to investigate an increasingly large number of genomic loci across many samples (O’Roak *et al.*, 2012). Optimized protocols resulted in the inclusion of unique molecular identifiers (UMIs) in the oligo sequence to increase data accuracy (Hiatt *et al.*, 2013), and machine learning algorithms were developed to improve coverage uniformity across smMIPs (Boyle *et al.*, 2014). These advances have primarily addressed the technical aspect of upfront smMIP design and data production, yet little progress has been made with respect to smMIP-data interpretation.

To date, a range of tools has been developed to detect somatic mutations from NGS (Xu, 2018). Many of those tools perform well in the experimental configurations for which they were designed. However, they cannot be generalized to different experimental formats and due to lack of specific functionalities, are expected to deliver sub-optimal results when smMIP sequencing is used. The need for a single standardized method tailored for smMIP-data analysis that efficiently provides accurate and reproducible results in a user-friendly, readable format remains.

To address this underserved need, we developed SmMIP-tools. Our method encases all the functionalities required for efficient and effective analysis of smMIP-derived sequencing information within a single software suite. Its only external dependency is on read alignment. Our approach to detect somatic variants comprises multiple features distinct from other variant callers and unlike other NGS error suppression techniques; it eliminates the need to sequence large experimental control cohorts to precisely define allele-specific error rates. The fidelity of error-corrected mutation calling is dramatically enhanced by analysing data from each read derived from sequencing read pairs, overlapping smMIPs and smMIP-library technical replicates when available. SmMIP-tools also outputs comprehensive variant annotations and include a unique variant flagging system to assist with ranking and prioritizing mutations associated with phenotypes of interest.

To demonstrate the real-world applicability of SmMIP-tools in genomic research, we chose to investigate genomic loci associated with both clonal haematopoiesis and myeloid malignancies. Clonal haematopoiesis is an age-related phenomenon defined by the expansion of blood cells with somatic mutations (Jaiswal *et al.*, 2014). It has been associated with an increased risk of all-cause mortality (Genovese *et al.*, 2014; Jaiswal *et al.*, 2014) cardiovascular disease (Jaiswal *et al.*, 2017) and the future development of leukaemia (Abelson *et al.*, 2018; Desai *et al.*, 2018). To establish these associations, previous studies required both labour-intensive and costly sequencing efforts. Instead, here we interrogated a similar genomic space using low-cost smMIP sequencing in conjunction with SmMIP-tools to benchmark and validate its performance (Supplementary Fig. S1).

## 2 Materials and methods

### 2.1 SmMIP-tools overview

**Fig. 1.**
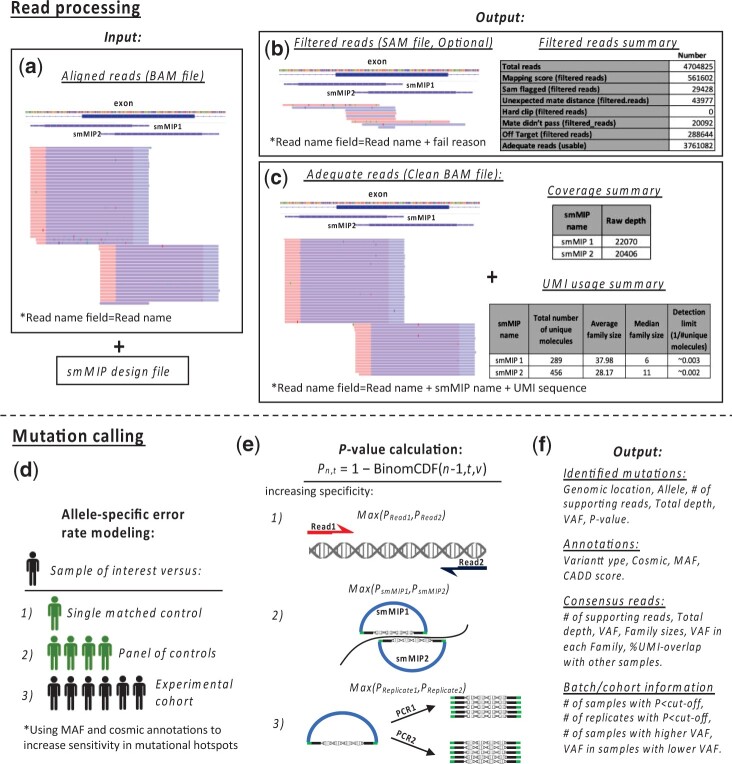
SmMIP-tools overview. (**a**) SmMIP-tools accepts single BAM files and a smMIP design file as inputs to assign reads to their probe-of-origin and filter problematic reads. (**b, c**) Following processing, SmMIP-tools outputs BAM files corresponding to filtered reads and reads that are appropriate for subsequent analysis. The output also includes three relevant summary files for the evaluation of sequencing library quality, coverage uniformity across smMIPs, and the number of unique molecules sequenced. (**d**) SmMIP-tools generates smMIP-level base call summaries (i.e. pileups) for SSCS and raw reads to determine non-reference alleles and their read support. These base call summaries serve as an input for the mutation calling algorithm. Error rates can be estimated from a single control, a larger cohort of controls or from the experimental cohort alone, without the need for additional control sample sequencing. (**e**) Allele-specific error rates are estimated by information derived from each strand, and overlapping smMIPs and technical duplicates when available. Let ‘*v*’ be the investigated allele’s fraction in the control cohort. Then, the *P*-value of seeing at least ‘*n*’ reads supporting the non-reference allele out of ‘*t*’ total coverage in the investigated genomic position in the sample of interest is P*n*, *t* = 1 − BinomCDF(*n*−1, *t*, *v*), where BinomCDF denotes the binomial cumulative distribution function. (**f**) The final output is a comprehensive report including the called variants as well as various annotations and flags that can be used to further prioritize mutation calls

SmMIP-tools effectively processes and analyses NGS data to report single nucleotide variants (SNVs), insertions and deletions (Indels) using several easy-to-execute steps. These steps principally rely on proper smMIP design and subsequent sequencing (Supplementary Fig. S2 and Supplementary Methods). Our method takes as input a read-alignment BAM file and a smMIP design file (Fig. 1a). The latter can be easily generated by MIPgen (Boyle *et al.*, 2014) or prepared manually. SmMIP-tools uses information concerning each probe and its target sequence to apply a set of filters and discard hard-clipped reads, reads with low mapping quality, paired reads with an unexpected insert size or improper alignment orientations (Fig. 1b). To confirm the proper structure of the remaining reads and to identify corrupted UMI sequences, linkage between reads and their precise probe-of-origin are generated. The final output contains quality control summary files concerning raw and consensus reads (Fig. 1c) and a BAM file with the remaining high-quality reads. UMI sequences and smMIP-of-origin identifiers are then included in each read’s header. In the following steps, SmMIP-tools uses the processed BAM file to generate probe-level base call summaries (i.e. pileups) that are subsequently refined by the software’s error-aware variant detection algorithm. Base call summaries for single-stranded consensus sequences (SSCS) are also generated simultaneously.

To call mutations, a multi-layered probabilistic approach is used to conduct allele-specific frequency comparisons between each sample of interest and either a single control or a cohort of control samples. Alternatively, SmMIP-tools can also precisely estimate allele-specific error rates without using dedicated controls by comparing a sample of interest to the remainder of the experimental cohort (Fig. 1d). Thus, SmMIP-tools can accommodate various user-defined experimental configurations to suppress errors. Prior knowledge concerning the location of common cancer mutations is also used to increase the sensitivity of detecting recurrently mutated alleles. To improve specificity, non-reference alleles are evaluated separately in each of the paired sequencing reads, in reads derived from overlapping smMIPs, and in technical replicates when available (Fig. 1e). The final output is a comprehensive report that includes the detected mutations, key variant annotations, information concerning consensus reads’ support, sequencing batch summaries and mutation flags, all of which are valuable for ranking and prioritizing variants (Fig. 1f and  Supplementary Methods).

A high-level comparison matrix emphasizing capability differences between SmMIP-tools and other existing software for processing and analysis of NGS-derived data is included in Supplementary Table S1.

### 2.2 Creation of read-smMIP linkages

To determine the probe-of-origin for every sequenced read pair, SmMIP-tools first searches for smMIPs whose targeted genomic loci, including the extension and ligation arms, substantially overlap with the genomic loci determined by the paired reads’ alignment to the genome (default 0.95, user-defined parameter). Once smMIP candidates are selected, the algorithm proceeds with the local alignment of each smMIP’s extension and ligation arms to the reads. The exact probe-of-origin is determined when both of its arms align in their expected positions (here, 4 nt from the reads’ extremes based on the length of UMIs). The location of UMI bases in each read of the pair (i.e. in the 5′ or 3′ end) is determined by the reads’ SAM flags. The number of UMI bases in each read is automatically determined from the user-provided panel design file. When the above alignment expectations are not met, the UMI will be considered unreliable, and the paired reads will not be used for further analysis concerning SSCS (Supplementary Methods).

### 2.3 Probabilistic modelling of error rates

SmMIP-tools uses the pbinom R function to calculate, for each observed allele in a sample of interest, a *P*-value reflecting the probability of obtaining a number equal to or higher than the observed number of non-reference supporting reads for the identical allele in a single matched control, a larger control cohort or with no controls. If the latter option is chosen, SmMIP-tools uses the entire cohort except for the sample of interest (and its technical replicate, if available) to estimate error rates. Sequenced alleles are annotated using the cellbaseR R package (Abdallah, 2020), and information concerning recurrent cancer mutations is leveraged to increase sensitivity at those positions. Accordingly, values of variant allele frequency (VAF) ≥ 0.05 (user-defined parameter) in recurrently mutated alleles are removed from error rate estimation. The allele frequency in all the other samples is set to their median value. To derive binomial probabilities, allele-specific error rates are determined as the sum of all the non-reference supporting reads in all the controls divided by the total number of reads covering the allele. In the event where there are zero non-reference supporting reads in the control sample(s) chosen, a pseudocount of one supporting read is added. This value is then evaluated against the number of non-reference supporting reads and the allele’s coverage in the sample of interest. If any allele is observed in both Read1 and Read2 it receives the higher *P*-value of the two models. This process is repeated for alleles that are covered by overlapping smMIPs and technical replicates

(all the other methods used in this study are provided as Supplementary Material).

## 3 Results

### 3.1 SmMIP-tools accurately links reads to their probe-of-origin to improve downstream data analysis

**Fig. 2.**
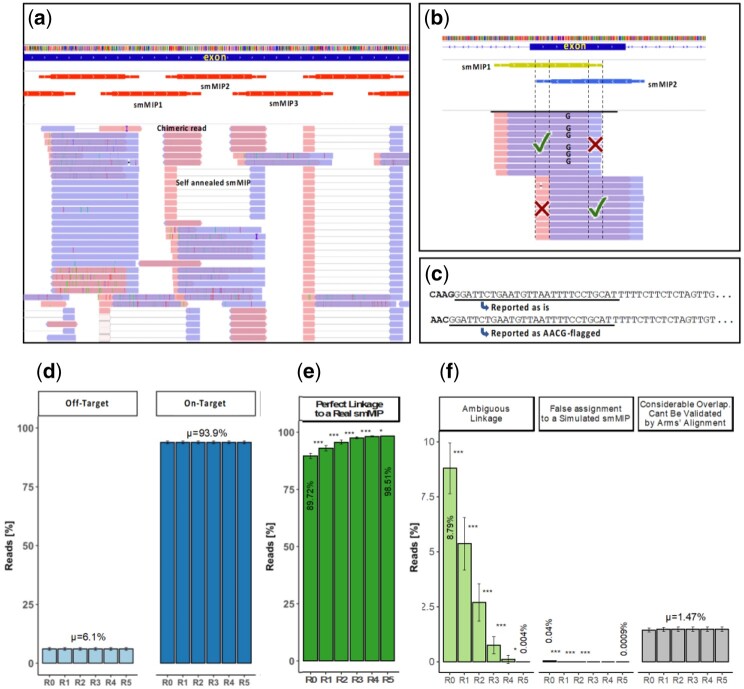
SmMIP-tools delivers accurate read-smMIP linkages allowing proper downstream analysis of data from complex smMIP panels. (**a**) Read-smMIP linkages support the identification and removal of error-prone sequencing library products. The example shows a representative chimeric read generated by one arm that belongs to smMIP1 and another to smMIP2, and a read without a target sequence generated from smMIP2. (**b**) Read-smMIP linkages help to suppress errors and define read sections that correspond to the target region, thus preventing mutation calling from their arm sequences. (**c**) Validating the correct position of the smMIP arm sequences in the expected reads layout helps identify UMI sequence errors to preserve high-quality data for subsequent analysis. (**d**) Average percentage of off-target and on-target reads. (**e**) Average percentage of perfect linkages of reads to a real smMIP. (**f**) Average percentage of incorrect linkages to a simulated smMIP. Error bars representing SDs for reads derived from the 16 interrogated samples. Paired Sample *t*-test: *P < 0.05, ***P < 0.001. R0 includes real smMIPs and all simulated smMIPs which may start or end at the same genomic location as real smMIPs. R3, e.g. includes real smMIPs but only simulated smMIPs that start or end more than three bases apart from real smMIPs’ start and end sites. R5, corresponds to >5 base difference

Identifying the correct smMIP arm sequences and validating their expected position in the sequenced read pairs is a critical processing step SmMIP-tools employs to improve downstream analysis of smMIP-derived data. Specifically, linking each sequencing read to its precise probe-of-origin (termed here, read-smMIP linkages) is essential to pinpoint self-annealed probes that lack target sequences and eliminate chimeric inserts generated by partially overlapping probes (Fig. 2a). Furthermore, read-smMIP linkages can help prevent mutation calling outside the target region of individual smMIPs, eliminate errors based on ambiguous calls in regions with overlapping smMIPs (Fig. 2b), and validate the UMIs’ sequence integrity in the expected insert layout (Fig. 2c).

To evaluate the performance of SmMIP-tools to correctly generate read-smMIP linkages, we sequenced 16 cord blood samples using smMIPs. We next interrogated the sequencing data considering the 284 real smMIPs used to generate it, together with a set of additionally designed simulated smMIPs (*n* = 22 299) that cover the same genomic loci (Supplementary Table S2) yet were not used during sequencing. The simulated probes were designed to include arms and target loci of variable length that can either partially overlap with those of the real smMIP, are fully encased within a real smMIP’s genomic insert, or extend beyond the real smMIP’s 3′ and/or 5′ end (Supplementary Fig. S3a). The entire set, including both the real and simulated smMIPs was designated as R0. From R0, we then generated additional smMIP subsets (R1–R5) by restricting the inclusion of simulated smMIPs based on how far their start and end position are from those of an overlapping real smMIP. Therefore, subset R1 retains only simulated smMIPs with a start and end that are at least 1 bp apart from the start and end of a real smMIP while excluding those with a distance ≤1 bp. The cumulative exclusion of simulated smMIPs continues for subsets R2–R5 (Supplementary Fig S3b). Based on these restriction criteria, the task of accurately assigning the correct probe-of-origin to each read and differentiating between real and simulated smMIPs is expected to be the most challenging for R0 and least challenging for subset R5.

A total of 25 353 671 read pairs generated from the 16 sequenced cord blood samples were subjected to read-smMIP linkage performance analysis using the algorithm embedded in SmMIP-tools’ code. On average, 6.1% of the reads could not be linked to any smMIP (real or simulated) and were considered ‘off-target’ due to insufficient overlap with the targeted loci (Fig. 2d). From the other 93.9% ‘on-target’ reads, SmMIP-tools assigned the correct probe-of-origin to 89.72% of the reads in R0 (Fig. 2e). In this set, 8.79% of the reads could equally be associated with more than one smMIP, and 0.04% were falsely assigned to a simulated smMIP (Fig. 2f). Performance was significantly improved when simulated smMIPs that start or end at an identical genomic base as real smMIPs were omitted (i.e. R1). In subset R5, 98.51% of the on-target reads were correctly assigned to a single real smMIP, and a negligible percentage of the reads were falsely assigned to a simulated smMIP (0.0009%). We noticed that sequencing and library amplification errors confound ambiguous or inaccurate assignment of reads to their probe-of-origin. On average, 1.47% of the on-target reads showed considerable overlap with the target locus yet due to errors failed validation. SmMIP-tools was designed to salvage such reads. Nevertheless, since their UMI sequence integrity might be compromised (Fig. 2c), such reads are flagged and not included in downstream analyses that consider SSCS.

Taken together, these results indicate that SmMIP-tools is capable of accurately constructing linkages between reads and smMIPs to address the technology’s constraints and prepare data derived from highly complex target panels, including those containing highly overlapping smMIPs, for more efficient and accurate downstream analyses.

### 3.2 SmMIP-tools deploys multiple layers of error-suppression techniques to enable highly accurate variant detection

**Fig. 3.**
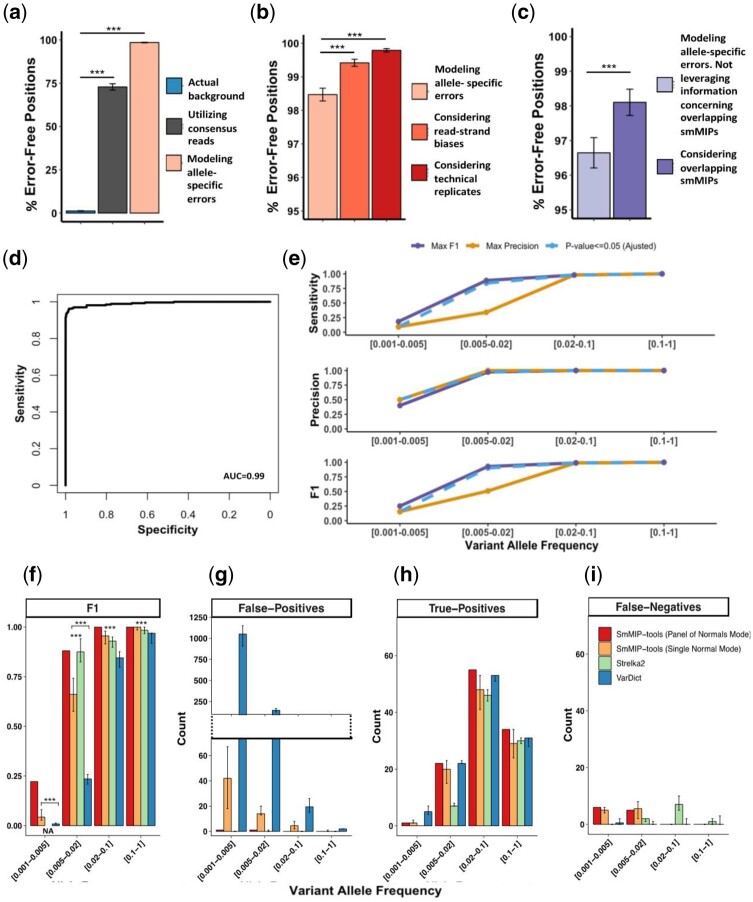
SmMIP-tools uses multiple approaches to suppress NGS-associated errors and accurately detect real mutations. (**a**) Percentage of error-free positions across the targeted genomic space before and following error suppression by SSCS assembly or by probabilistic error rate modelling. The average error background in the cell line DNA mixes is shown for comparison. (**b**) Percentage of error-free positions following probabilistic error rate modelling and consideration of information derived either from paired reads or technical replicates. (**c**) Percentage of error-free positions following probabilistic error rate modelling alone versus the additional consideration of overlapping smMIPs. Here, positions covered by overlapping smMIPs were considered to derive the percentage of error-free positions. (**d**) Receiver operating characteristic curve indicating the performance of SmMIP-tools to detect real mutations among the high background of NGS-associated errors. (**e**) Accuracy (precision, sensitivity and F1 score) is shown across different VAF ranges. Coloured lines represent the results obtained when the core algorithm was set to achieve maximum F1, maximum precision or when the default *P*-value cut-off (0.05) was used. (**f–i**) Performance evaluation of mutation calling methods. Bars represent median values obtained following mutation calling with each of the 16 cord blood samples used separately as controls. Error bars represent the maximum and minimum obtained values. Mann–Whitney test for the selected comparisons: ***P < 0.001

SmMIP-tools incorporates multiple error-suppression techniques (Fig. 1d–f) to distinguish real mutations from NGS-associated errors and suppress false-positive calls. To benchmark their use, we first constructed high confidence lists of true and false-positive mutations by bulk sequencing of eight blood cancer cell lines (Supplementary Methods and Table S3). DNA from the different cell lines was then mixed to generate six separate pools containing varying concentrations of each cell lines’ genomic material (Supplementary Table S3). Each mix was sequenced twice to enhance error suppression through the use of information derived from technical replicates. In each of the 12 sequenced libraries, we counted the number of error-free positions, defined as positions in the interrogated genomic space represented exclusively with reference alleles, before and after applying error suppression techniques. Both the consensus reads assembly (Supplementary Methods) and the probabilistic error rate modelling techniques (Supplementary Fig. S4), delivered significant levels of error suppression as indicated by the sole presence of reference alleles in 72.83% and 98.47% of the investigated genomic positions, respectively, compared with an average of 1.22% before error correction (Fig. 3a). Error suppression using the error rate modelling approach was further augmented when information derived from separate read-strands or technical replicates was incorporated (Fig. 3b). Significantly improved error suppression was also observed when alleles covered by overlapping smMIPs were evaluated against error models derived from each smMIP independently (Fig. 3c). Next, we evaluated SmMIP-tools’ performance in differentiating between real mutations and errors after intersecting all of the error suppression techniques mentioned above. We required at least one SSCS in each strand, and for error rate modelling, we used data derived from separate read-strands, overlapping smMIPs and technical replicates. SmMIP-tools accurately identified the real mutations among the high background of NGS-associated errors, as evident by a near-perfect trade-off between sensitivity and specificity (Fig. 3d). Sensitivity and precision remained high down to a VAF of 0.005, only decreasing to a lower limit of 9.1% sensitivity and 50% precision for mutations detected in the 0.001 < VAF < 0.005 range (Fig. 3e).

Finally, we sought to compare the performance of SmMIP-tools with other established variant callers that are also capable of both SNV and Indel detection. Somatic mutations (*n* = 24) that were detected in the bulk cell line sequencing served as the ‘ground truth’ against which we evaluated performance (Supplementary Table S3). SmMIP-tools, VarDict (Lai *et al.*, 2016) and Strelka2 (Kim *et al.*, 2018) were all tested using their default settings (Supplementary Methods). Both VarDict and Strelka2 demonstrated inferior results across multiple VAF ranges as compared with SmMlP-tools (Fig. 3f). Interestingly, these results originated from a different suboptimal balance between false-positive (Fig. 3g), true-positive (Fig. 3h) and false-negative calls (Fig. 3i). Most notable was VarDict’s increasing number of false-positive calls. On the other hand, Strelka2 successfully eliminated false-positive calls yet failed to report many true-positive mutations below VAF of 0.02. Consequently, the overall accuracy, measured as F1-scores, of the reports generated by the two other commonly used methods, significantly suffered compared with that of SmMIP-tools (Fig. 3f).

Errors are key confounding factors for sensitive detection of low-frequency variants by deep sequencing (Abelson *et al.*, 2020; Ma *et al.*, 2019). These analyses show that SmMIP-tools’ multi-layered error suppression techniques enable accurate differentiation between real mutations and abundant NGS acquired noise across a wide VAF range.

### 3.3 SmMIP-tools detects new deleterious mutations in a comparative analysis with diagnostic test results

**Fig. 4.**
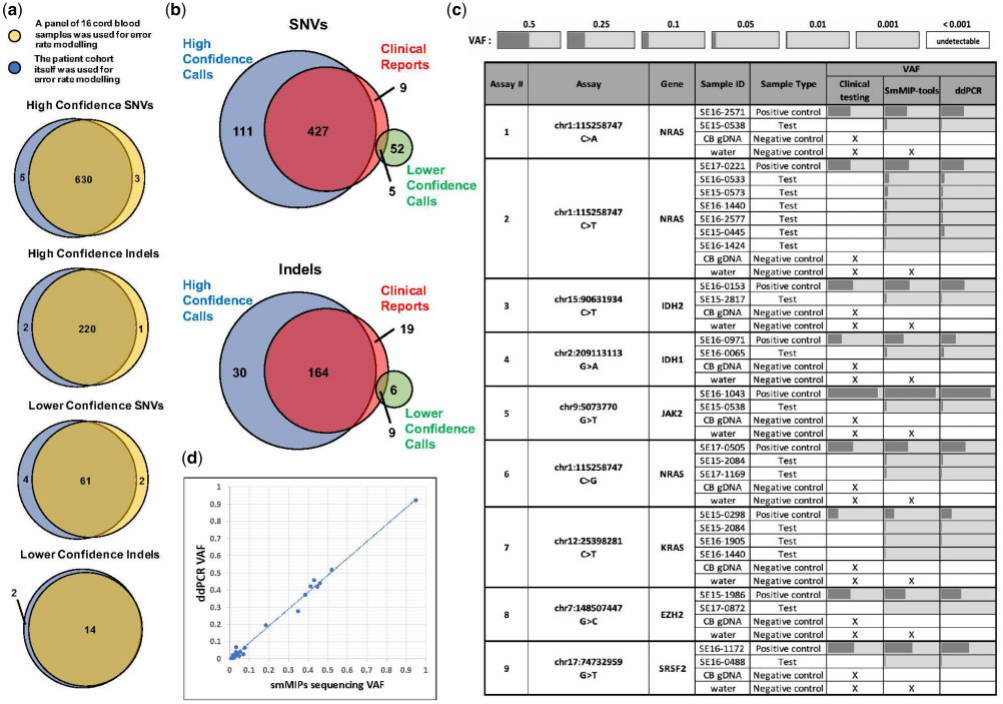
SmMIP-tools enables high confidence detection of pathological variants. (**a**) Venn diagrams representing the number of mutations called by two different approaches for error rate modelling and their overlap. (**b**) Venn diagrams for SNVs and Indels illustrate the number of mutations that are shared between SmMIP-tools output and the clinical genetic reports for overlapping panel regions, as well as the number of mutations that differ between the two sources. (**c**) Validation by ddPCR of 17 high confidence mutations identified by SmMIP-tools yet did not pass detection using clinical diagnostic testing. White background indicates that the variant was not reported while light grey background indicates the variant was detected above the ddPCR detection limit (VAF > 0.001). Dark grey bars represent the VAFs. X, not tested. (**d**) Scatterplot showing a strong linear correlation (Pearson *r* = 0.996, *P*-value = 9.81 × 10^−7^) between VAFs calculated by ddPCR and those derived from the SmMIP-tools output for the high confidence SNVs interrogated in the test samples and positive controls

To test the performance of SmMIP-tools in a real-world, clinically relevant cohort, we undertook comparative analysis between SmMIP-tools output and clinical genetic testing in patients diagnosed with myeloid malignancies. Re-sequencing of 168 samples from 162 patients using smMIPs was conducted in technical duplicates. Clinical reports were available for 135 of the patients (Supplementary Table S4). We first tested the performance of SmMIP-tools using either a dedicated cohort of controls for error rate modelling or alternatively, using only the sequencing data derived from the patient samples in the experimental cohort itself. Two mutation categories termed ‘High Confidence’ and ‘Lower Confidence’ were evaluated based on SmMIP-tools’ generated flags (Supplementary Methods); both showed highly reproducible results between the two error rate modelling approaches (Fig. 4a).

By leveraging information concerning the reads’ smMIP-of-origin, their duplication level (i.e. family size), and UMI sequences, we found that sample index-misassignment (also termed as ‘index-hopping’) is a likely source of several potentially stochastic errors (Supplementary Table S5). Importantly, these included high VAF, clinically relevant mutations that passed detection by probabilistic error rate modelling (Supplementary Fig. S5). Yet, these potentially false-positive variants were detected only by single reads (i.e. singletons with SSCS family size = 1). Moreover, all of the singletons’ UMIs pointed to identical reads represented in multiple other samples in the sequenced cohort. Further supporting index-hopping, nine of the 23 variants flagged by SmMIP-tools are common single nucleotide polymorphisms suggesting an abundant source of reads that were potentially misassigned. These nine variants were detected with atypical VAF far below the expected ∼50% for germline mutations.

For comparison between the patients’ clinical genetic testing and SmMIP-tools’ output, we used the experimental cohort for error rate modelling and considered only genomic loci covered by both sequencing panels (Supplementary Table S2). Overall, 95.6% of the somatic SNVs and Indels detected using the clinical pipeline were also detected by SmMIP-tools (Fig. 4b and  Supplementary Table S4). Of these, 97.7% were in the ‘High Confidence’ category. Technical issues unrelated to SmMIP-tools were identified to be the primary reason for the 28 variants that were missed (Supplementary Table S4). These include, e.g. deletions that failed recognition by the external read-alignment algorithm used (Supplementary Fig. S6). It is possible that slightly modified smMIP design or using other alignment tools that better support gapped alignment might improve detection of some Indels, such as those reported here. Importantly, SmMIP-tools detected an additional 111 high confidence SNVs (additional 25.2%) and 30 Indels (additional 15.6%) that did not pass detection by clinical testing (Fig. 4b and  Supplementary Table S6). The 111 newly discovered SNVs had an average scaled Combined Annotation Dependent Depletion score (Rentzsch *et al.*, 2019) of 29.7, indicating a striking enrichment for mutations predicted to be deleterious. A subset of these newly detected high confidence deleterious SNVs was validated by digital droplet PCR (ddPCR), with a 100% success rate (Fig. 4c and  Supplementary Methods). A significant correlation was observed between the VAFs obtained by smMIP sequencing and the ddPCR results (Fig. 4d).

These analyses shows that sequencing of healthy control samples for error suppression may not be necessary when large patient cohorts are sequenced and collectively emphasize the potential of using SmMIP-tools in conjunction with low-cost smMIP sequencing for clinical testing.

## 4 Discussion

In this article, we present a computational method designed to ease, improve and standardize the necessary steps involved in smMIP-derived data analysis. SmMIP-tools is specifically tailored to address the high error rates associated with amplicon-based sequencing and support the implementation of cost-effective molecular inversion probes-based NGS.

By linking each sequence read to its probe-of-origin, SmMIP-tools can identify and filter error-prone reads, such as chimeric reads or those derived from self-annealing probes that are uniquely associated with smMIP-based sequencing. Prevention of mutation calling outside of the smMIP target region and identification of corrupt UMI sequences are two other essential deliverables of read-smMIP linkages. Conclusively, we observed a significant contribution of read-smMIP linkages for NGS-error suppression. We further inform best practices for smMIP panel design by demonstrating SmMIP-tools’ ability to resolve complex datasets consisting of highly overlapping smMIPs.

Defining the absolute ground truth is a major challenge when reporting mutations from NGS data (Koboldt, 2020). Errors that arise during library preparation and sequencing are abundant and can easily obscure real mutations. Following data processing, SmMIP-tools implements a versatile error rate modelling approach to calculate a *P*-value for every non-reference allele observed in the data to reflect the probability of a false observation. Error suppression is enhanced by comparing observations derived from separate sequencing read pairs, overlapping smMIPs and technical replicates. Moreover, SmMIP-tools error-modelling approach is versatile compared with many other variant callers that are capable of addressing only a single experimental configuration. We show that SmMIP-tools can derive remarkably comparable results using either a dedicated cohort of controls or by leveraging data across the experimental cohort itself for probabilistic modelling. This unique capability enables prospective users to reduce the cost and labour associated with control cohort sequencing. Using a single control sample is also a viable option. However, in such experimental design, applying more stringent analytic parameters, such as lower *P*-value and higher VAF cut-offs are recommended to better control for the large number of observed stochastic NGS errors. It is important to note that while SmMIP-tools supports the use of consensus sequences, some errors such as those derived from oxidative DNA damage at guanine nucleotides are better suppressed using double-stranded sequencing (Salk *et al.*, 2018). By using smMIPs and intersecting multiple layers of error-suppression, our analysis with known ground-truth mutations demonstrated a near-perfect performance down to VAF of 0.005.

Since smMIP sequencing often involves large cohorts (Mamanova *et al.*, 2010), we designed a comprehensive variant flagging system to support the authenticity of variants by leveraging information derived from the entire sequencing run. In addition to *P*-values, SmMIP-tools analyses batch-related information, including the VAF of the called alleles in other samples, the number of instances in which the identical alleles were observed with a higher VAF and the number of additional samples in which the allele was detected above the background sequencing noise. The unique ability of SmMIP-tools to leverage batch information help to identify suboptimal error rate modelling events for specific alleles, at positions with elevated error rates that may require further validation by orthogonal methods. Another type of data that is critical to prioritize mutation calls is batch-related UMI information. Conventionally, UMIs are used to identify PCR duplicates to generate consensus sequences with lower error rates. In addition to the creation of SSCS, here we provide a novel *in* *silico* approach employing UMIs to address mutation calls that potentially arose due to sample index misassignment. It is important to note that unlike our strategy to mitigate the negative consequences of index misassignment, the use of non-combinatorial dual sample indexes can allow direct identification and removal of swapped reads (Costello *et al.*, 2018). Nevertheless, sequencing with non-combinatorial dual-indexed adapters substantially limits multiplexing capability which is a major strength of smMIP-based sequencing.

The implications of cost-effective, highly accurate and sensitive mutation detection are far-reaching (Karlovich and Williams, 2019). To establish proof-of-principle for the utility of the high-quality analysis provided by SmMIP-tools, we used smMIPs to resequence a large cohort of patients diagnosed with myeloid neoplasms. We show the ability of SmMIP-tools to not only detect variants previously reported by clinical testing but also reveal, with high confidence, additional deleterious variants. These findings illustrate the potential utility of deploying SmMIP-tools in clinical settings as a more cost-effective and sensitive alternative for genetic testing. Furthermore, reliable variant discovery at low VAF enables subclonal detection that when paired with longitudinal sequencing might help to guide therapies in real-time where actionable targets exist. Early detection with subsequent intervention may also be possible and population-based association studies, such as those done for clonal haematopoiesis, become more feasible.

Overall, this study demonstrates the untapped potential of utilizing SmMIP-tools, in conjunction with smMIP-based sequencing, to deliver superior and more accurate data at a fraction of the cost compared with other more labour-intensive sequencing approaches. We anticipate that SmMIP-tools will greatly facilitate broad applications of low-cost, targeted NGS, enabling the use of a single computational method instead of an alternative ensemble of unspecialized software to easily derive accurate results from smMIP data. Improving analytical accuracy and easing code execution will significantly influence data quality and the accessibility of the technology to computational and non-computational labs alike, pushing large-scale genetic research and personalized medicine forward. 

## Supplementary Material

btac081_supplementary_data
