# Supplementary material for: SmMIP-tools: a computational toolset for processing and analysis of single-molecule molecular inversion probes-derived data
Source: Bioinformatics. 2022 Feb 12;38(8):2088–95. doi: 10.1093/bioinformatics/btac081 (PMC9004652; doi:10.1093/bioinformatics/btac081)
Supplement: btac081_supplementary_data [file btac081_supplementary_data.zip › Supplementary File 1.pdf]

Supplementary Methods:

SmMIP panel design to maximize error identification and suppression

SmMIPs were designed using MIPgen (Boyle *et al.*, 2014) to include dual UMIs, each four bases in length, at each side of the smMIPs' backbone sequence. This UMI design allows collapsing of reads to the single smMIP-captured molecule from which they derived. Up to 65,536 unique molecules per smMIP targeted loci can be detected. We also designed dual sample indices, each eight bases in length, on the forward and reverse PCR primer to enable the high multiplex sequencing conducted in this study. All the oligos sequences that are needed for successful smMIP sequencing are illustrated in Fig. S2. In this study, we targeted hotspot variants and exons known to be commonly mutated in the blood during ageing as well as in patients with myeloid malignancies (Supplementary Table S2). To maximize the error-correction capabilities of SmMIP-tools, we followed two guiding principles. First, the read length of the intended sequencing platform of interest was considered to help determine the optimal target sequence length that allows every smMIP target to be sequenced by both paired-end sequencing reads thoroughly. Second, when a relevant genomic region could not be covered entirely by a single smMIP, we designed additional overlapping smMIPs to tile the region. No preference was set to design overlapping smMIPs on opposite DNA strands. Based on analysis of simulated data, smMIPs were designed to align >5 bases apart from the start and end site of their overlapping smMIPs to eliminate ambiguous read-smMIP linkages.

SmMIP-Library preparation and sequencing

SmMIP libraries were generated as described elsewhere (Hiatt *et al.*, 2013; Cantsilieris *et al.*, 2017) with some modifications. Phosphorylation of the smMIP pool was performed using a smMIP phosphorylation reaction previously described (Cantsilieris *et al.*, 2017). Once phosphorylated, the smMIP pool was diluted such that 4ul would achieve a 1:1000 DNA:smMIP molecular ratio in subsequent target capture steps when using 5ul of DNA prepared at 20ng/ul, for a total input of 100ng. The probe capture reaction, including the probe hybridization phase, gap-fill and ligation phase, as well as exonuclease treatment, were performed as previously described (Hiatt *et al.*, 2013). PCR amplification of exonuclease-treated smMIP captured products were also performed as previously described (Hiatt *et al.*, 2013) with the exception of the annealing temperature being increased from 15 to 30 seconds for each PCR cycle, followed by an additional final incubation of 72°C for 2 minutes and infinite hold at 4°C. Technical duplicates of PCR amplified libraries were generated for each sample, using unique combinations of barcoded PCR primers. Once generated, sample libraries in each 96-well plate were pooled by volume. Each pool was quantified using Qubit (High-Sensitivity dsDNA Assay, Thermo Fisher Scientific) and underwent a PCR clean-up using MinElute columns (Qiagen). Up to 5ug of input was used per column. Elution was made with 15ul of molecular-grade water. Each pool was subjected to quality control steps, including quantification and fragment analysis on the BioAnalyzer 2100 high-sensitivity assay (Agilent). All the different plate-derived pools were then pooled together by concentration taking into consideration the number of samples in each plate. This final pool then

underwent size selection using PippinHT (Sage Science) to mitigate the carryover of smaller and larger products suspected to be self-annealed smMIPs without target sequences and complex oligo hybrids, respectively. Lastly, pooled and size-selected multiplexed smMIP libraries were subjected to sequencing. Bulk cell line libraries were sequenced on the NextSeq using the Mid-Output kit (Illumina). Cord blood, cell line DNA mixes and patient libraries were sequenced using a single NovaSeq SP flow cell (Illumina). 10% PhiX was used to ensure sufficient library complexity.

Assembly of single strand consensus sequences

SmMIP-tools uses the R-package Rsamtools (Morgan M, Pagès H, Obenchain V, Hayden N . Rsamtools: Binary alignment (BAM), FASTA, variant call (BCF)) to generate base call summaries which are subsequently used to calculate allele frequencies across the target panel. SmMIP-tools extend Rsamtools functionalities by generating base call summaries for every UMI-smMIP combination. To minimize information loss, the minimum depth parameter was set to one read, the minimum read mapping quality was set to 50 and the base quality cut-off to 10. At each genomic position, the most frequent allele was determined based on a 70% threshold as suggested previously (Kennedy *et al.*, 2014). We did not restrict read family size since this information is being captured in the final SmMIP-tools report. The final report allows complete evaluation of the data and is used to flag and rank reported alleles.

Scalability of SmMIP-tools

We ran SmMIP-tools read-processing algorithms to assess computational scalability. Performance was measured as the time to process 1,000,000 paired-end reads (2,000,000 total) using a laptop equipped with 32 Gb total RAM and four 2.9 GHz, Intel i9 cores. Specifically, mapping reads to their corresponding smMIPs and validation of the mapping by local smMIP arms alignment (read-smMIP linkages), creating QC summary files, read filtering, writing a clean BAM file, and generating both smMIP-level raw and consensus base-call summaries was completed within 51 minutes. With increasing sample size the use high-performance computing (HPC) clusters will be required. SmMIP-tools is capable of multithreading to make a better use of the available computational resources.

Derivation of mutation lists for SmMIP-tools benchmarking using cell line DNA mixes

To construct lists of high confidence errors and mutated alleles to benchmark the SmMIP-tools mutation calling algorithm, we first sequenced the DNA of eight cell lines, namely ME-1, Kasumi-1, KG-1A, U937, SU-DHL-10, OCI-AML3, OCI-AML-22, and OCI-AML-8227. Each cancer cell line was sequenced in four replicates. We called mutations in each of the 32 samples independently using the experimental cohort itself for allele-specific error rate estimations. That is, allele frequencies in each investigated sample were evaluated against those observed in all the samples of the other cell lines. Altogether, we identified 44 true-positive SNVs and four insertions (Supplementary Table S3). True-positive status was determined if a mutation: 1) Received a P-value<=0.05, 2) was found in at least 3 out of 4 replicates with VAF>=0.02, and 3) was supported

by at least 1 SSCS from each strand (Read1 and Read2). We removed mutations if they were shared by more than three different cell lines to prevent the list from being populated with a large number of uninformative, prevalent germline mutations. The status of alleles assigned with a P-value<=0.05 in individual samples but did not pass the additional criteria above was determined as "Uncertain". Those alleles were eliminated from benchmarking (Supplementary Table S3). Every other non-reference allele in the remaining interrogated genomic positions was considered false-positive if seen in the follow-up sequencing of the DNA mixes. For cell line mixes data analysis using SmMIP-tools, a minimum of one SSCS in each strand was required. A control cohort of 16 umbilical cord blood samples was used to derive probabilistic error rate models. The distribution of the binomial p parameter assigned to models used to call mutations across the entire target panel are shown for both the plus and minus strands (Supplementary Fig S4). A P-value threshold of 0.05 was set to suppress errors following Bonferroni correction for multiple tests. True-positives were removed to calculate the percentage of error-free positions. To ensure a fair, unbiased comparison with SmMIP-tools, VarDict (Lai *et al.*, 2016) and Strelka2 (Kim *et al.*, 2018) were used according to their recommended default parameters. We conducted multiple iterations of mutation calling. In each of the 16 iterations, a different cord blood sample was served as control and the output was merged into a single file. We also ran SmMIP-tools in a single-normal mode. For all the methods, only variants that were reported in both the technical replicates from each investigated cell line DNA mix in each iteration were considered to determine the accuracy of each tool's mutation-calls report. True-positives, false-positives and false-negatives were defined based on each tool's filter tags, and the mutations' presence or absence in the list of the ground-truth cell line somatic mutations (Supplementary Table S3). Explicitly, a ground-truth allele that received the filter tag "PASS" using Strelka2 or VarDict was considered a true-positive call. If it received a different filter tag it was considered as a false-negative. We compared reports, therefore, ground-truth alleles that were not included in one tool's report were not counted as false-negatives.
Mutations that received the filter tag "PASS" yet were not in the list of ground-truth somatic mutations were considered false-positives. For SmMIP-tools we asked for a P-value<=0.05 (corrected) and no additional flags.

Mutation categories

By default, SmMIP-tools generates a report that includes baseline mutation calls defined as those mutations detected with a Bonferroni corrected P-value lower than or equal to the user-defined P-value cutoff. In this study, we used a cutoff of 0.05. The number of tests used for Bonferroni correction is equal to the total number of alleles that can potentially be detected using the smMIP target panel. These include "A", "C", "T", "G", "-" (deletion) and "+" (insertion) at every targeted genomic position. To enrich the baseline calls for somatic and pathogenic mutations, we removed common single nucleotide polymorphisms with a minor allele frequency (MAF)>0.01 as well as mutations assigned with the "potentially germline" flag that was determined based on MAF cutoffs of 0.001<=MAF<0.01 (user-defined parameters). SNVs with the "potentially germline" flag were kept only if they have been reported in the COSMIC database (Tate *et al.*, 2019). We also removed

SNVs that were assigned the "likely benign" flag. Those include mutations with one of the following annotations: "synonymous variant", "intron variant", "splice region variant" (rather than "splice acceptor/donor variants"), "non-coding transcript exon variant", "5 prime UTR variant", or "3 prime UTR variant". For Indels, we removed those that fall entirely within intronic regions. The report generated by the SmMIP-tools mutation calling algorithm also includes additional information concerning consensus reads and details about how baseline called alleles are being represented in other samples in the sequenced cohort. We used this information to derive two categories for mutation ranking, namely "High confidence" and "Lower confidence". 1) High confidence mutations were determined as those mutations that: a) Received a P-value that is lower than or equal to 0.05. b) Are enriched for somatic and pathogenic mutations as described above. c) Had no flags indicating possible technical issues that may interfere with their detection (i.e., the flags: "Cannot be supported by both Read1 and Read2", "Cannot be supported by all overlapping smMIPs", "Cannot be supported by both technical replicates", "No SSCS support in one of the replicates", "No SSCS support at all", "No SSCS support in either Read1 or Read2 in at least one of the replicates"). d) Had no flags indicative of potential false positives (i.e., the flag "Potential index hopping"). The "Potential index hopping" flag is generated when variants are supported only by singletons reads (family of size 1) and when all of those reads' identities are also observed in other samples in the sequenced cohort. Reads' identities are defined based on the variant that the reads report, their UMI and the smMIP used to generate them. e) Did not have the "VAF Warning" flag. The absence of this flag indicates that the mutation was detected with the highest VAF in the reported sample and that its VAF is at least 2-fold (user-defined parameter) higher as compared with all the other samples in the entire cohort. Of note, by default, the algorithm that generates this flag permits that a single sample with a lower VAF will not reach the 2-fold VAF difference and was designed to account for true recurrent cancer mutations detected as baseline calls in the cohort. 2) Lower confidence mutations were determined as those mutations that received flags indicating potential technical issues that could have interfered with their detection. Those include mutations that were detected only by Read1 or Read2 or only in a single smMIP where overlapping smMIPs are present. Of note, optimal smMIP design where every targeted base is sequenced twice, once with Read1 and once with Read2, can reduce the number of lower confidence calls. Other mutations in this category are mutations that were not detected in both the technical replicates due to low coverage anomalies in one of the replicates. A minimum coverage of 10x from each read (Read1 and Read2) (user-defined parameter) was required to consider strand-derived information for P-value calculations. When the coverage does not reach the set threshold for any mutation call, a detailed "Low coverage" flag is generated. More specifically, we defined and reported lower confidence mutations if: a) They were detected in at least 50% of the available detection options mentioned above (i.e., overlapping sequencing reads, overlapping smMIPs and technical replicates). For example, if a mutation was detected in both Read1 and Read2 but in only one replicate, where overlapping smMIPs do not exist, it will be reported since it was detected in 50% of the possible detection options. If a mutation that is covered by a single smMIP was detected with only Read1 in only one replicate, it would not be reported since it was detected in only 1 of

the 4 possible options. b) There were not more than five other samples in the entire sequenced cohort where those mutations received a P-value<=0.05. c) There were no other samples that were detected with those mutations with a higher VAF (only relevant to mutations that lack a COSMIC ID).

Patient cohort

All patients referred to the MPN/leukemia program at the Princess Margaret Cancer Center were approached for targeted sequencing as part of the Advanced Genomics in Leukemia (AGILE) prospective study (Alduaij *et al.*, 2018). All biological samples were collected according to procedures approved by the Research Ethics Board of the University Health Network (01-0573.28) and were viably frozen in the Princess Margaret, Leukemia Tissue Bank. Written informed consent was obtained from all patients in accordance with the declaration of Helsinki.

Clinical reports

DNA samples extracted from peripheral blood or bone marrow were used for NGS. Sequencing was performed using the TruSight Myeloid Sequencing Panel (Illumina) and run on the MiSeq Illumina platform as previously described using a protocol that was validated by the University Health Network Advanced Molecular Diagnostics Laboratory (Thomas *et al.*, 2017). Briefly, 54 genes implicated in myeloid malignancies were profiled using amplicon-based library preparation (Supplementary Table S2). Data analysis and quality assessment for reporting of SNVs and short Indels was performed using NextGene v.2.3.1 (SoftGenetics, State College, PA). Detected variants were then annotated using the WHO classification and diagnostic criteria as previously reported (Barbui *et al.*, 2018) to differentiate between variants of unknown significance and those that are oncogenic. Oncogenic variants detected at coverage >100X, with VAF>5%, were included for subsequent investigation. Known hotspot variants detected below these thresholds were verified using either Sanger sequencing or ddPCR. Detection of recurrent NPM1, Indels, and FLT3 internal tandem duplications was performed using fluorescent polymerase chain reaction (PCR) followed by fragment analysis. Variants that repeatedly occurred in >10% of cases, excluding mutations in hotspot regions or variants previously reported in the literature, were filtered out.

Digital droplet PCR

We used ddPCR to validate a subset of SNVs that were detected as “High Confidence” mutations by SmMIP-tools but were not reported in the clinical sequencing reports. Nine ddPCR assays were designed to target oncogenic SNVs spanning a VAF range of 0.008-0.077, as determined by SmMIP-tools, in 17 patients (n=1 VAF<0.01, n=5 0.01<VAF<0.02, n=8 0.02<VAF<0.05, n=3 0.05<VAF<0.08). Wild type and mutant specific PrimeTime LNA qPCR probes were conjugated to HEX and FAM fluorophores, respectively (Integrated DNA Technologies). 20ng of DNA, originally used for clinical sequencing and re-sequencing using smMIPs, was subjected to ddPCR assays in a 96-well plate according to the manufacturer’s protocol (Bio-Rad). Droplets were generated and read using the QX200 ddPCR system (Bio-Rad) to detect the absolute number of

reference (HEX) and non-reference (FAM) alleles. VAF was calculated as the number of FAM captured targets over the total number of targets (FAM+HEX). As previously reported (Shlush *et al.*, 2017), variants were considered positive if >3 droplets were captured in the mutant (FAM) channel with a final VAF>0.001. Samples with the same mutations detected by both the clinical pipeline and SmMIP-tools were used as positive controls. Cord blood DNA was used as a negative control and water was used as a blank. ddPCR assay conditions and associated primer and probe sequences are supplemented (Supplementary Table S7).

Supplementary Figures:

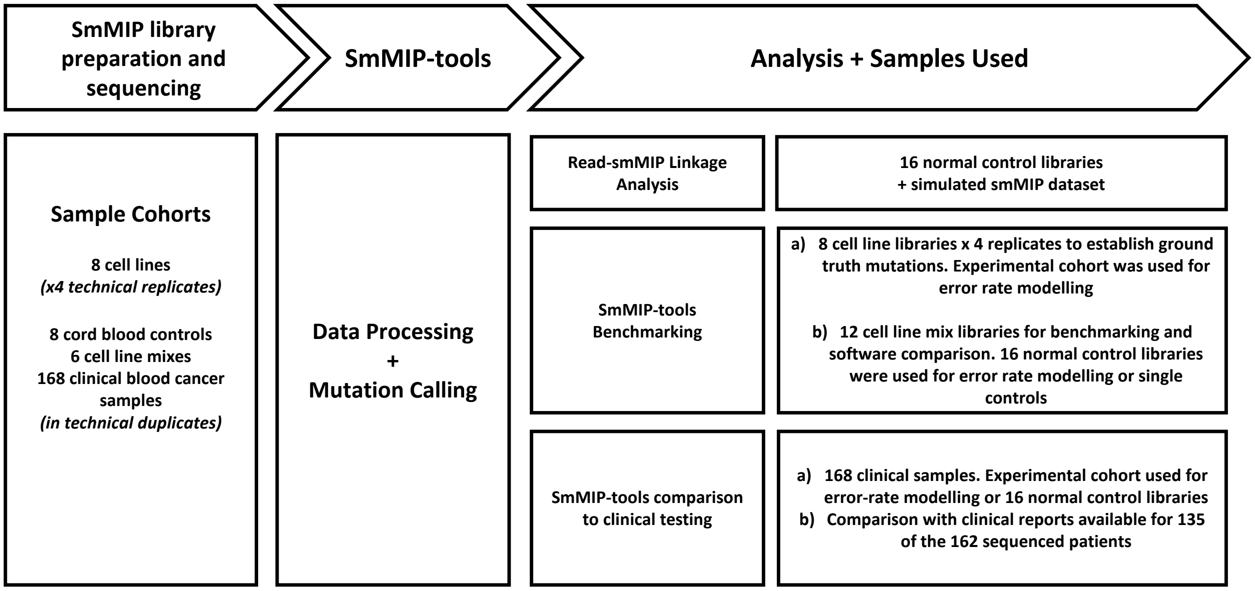

Figure S1. Overview of the sequencing experiments and analysis performed.

smMIPs and primers design

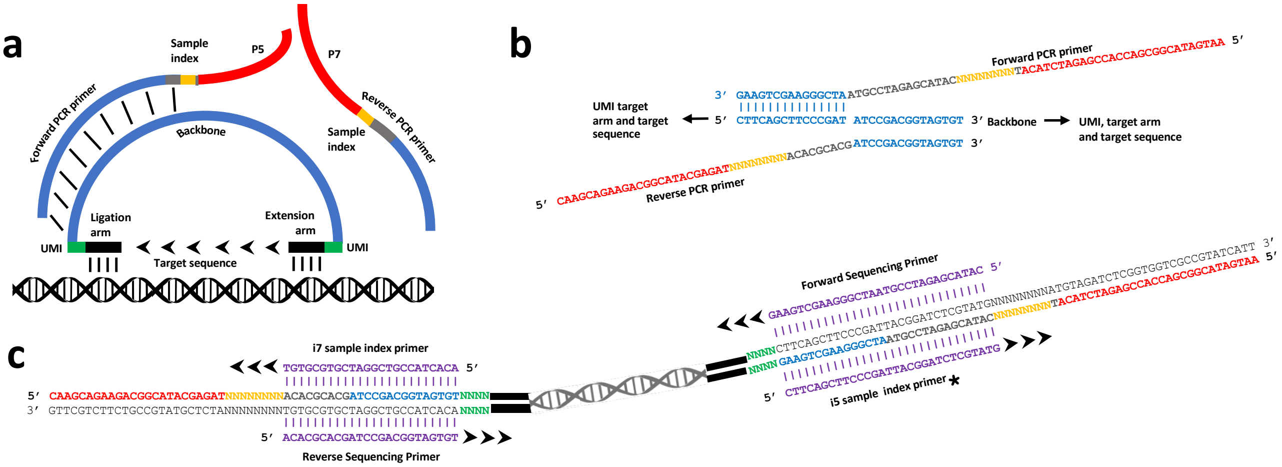

Figure S2. Proper smMIP and sequencing primer design. **a** General layout of a smMIP. One can improve smMIP panel design by accounting for the sequencing platform intended for use with respect to its output read length. Assuring that each of the paired reads thoroughly interrogates its corresponding target locus will maximize the coverage at each base and support the suppression of errors that preferentially occur on one of the two amplified cDNA strands. **b** Backbone and PCR primer sequences and annealing sites. **c** Library composition and sequencing primer sequences. *the i5 sample index primer is only required for select sequencing platforms. >>> indicates the direction of extension.

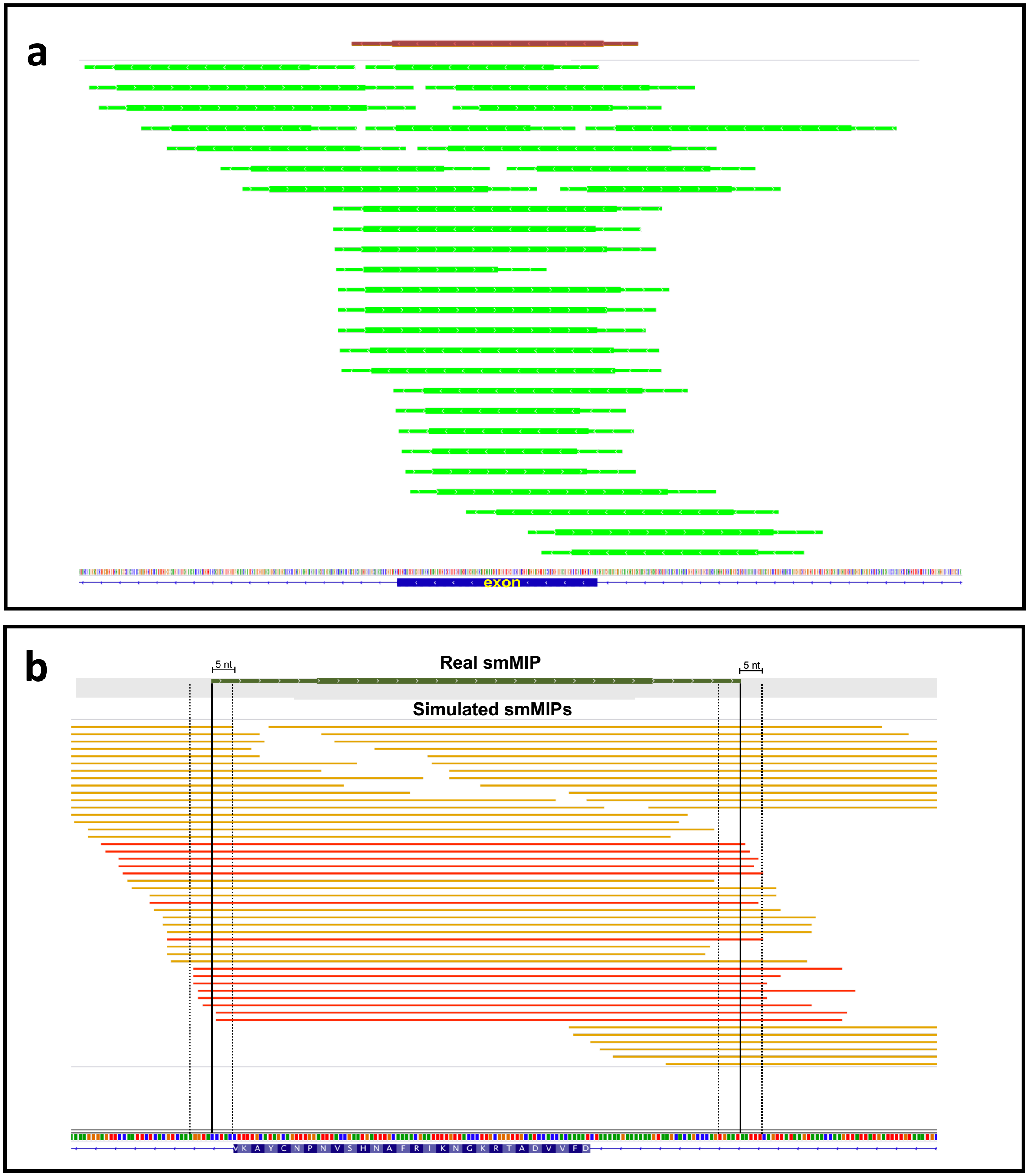

Figure S3. Simulating smMIPs to assess the performance of read-smMIP linkage generation. **a.** Simulated smMIPs (green, n=22,299) were designed in the proximity of real smMIPs that were used for sequencing (brown, n=284). A simulated smMIP can either overlap with a real smMIP, be smaller and included within the genomic region belonging to that of a real smMIP or be larger and flank the entire genomic region of a real smMIP. Broad and narrow lines represent each smMIPs' target region and target arms respectively. **b.** The further the start and end genomic positions of simulated smMIPs are to those of real smMIPs, the easier it is to accurately determine the reads' origin. While all the simulated smMIPs (red and yellow) were included in the R0 subset, red smMIPs, as this example shows, were removed to generate the R5 subset. Red color represents simulated smMIPs with at least one of their ends being <=5nt to that of the real smMIP's ends. Solid vertical lines represent the start and end positions of the real smMIP while dashed vertical lines represent distance of 5 nucleotides from these ends.

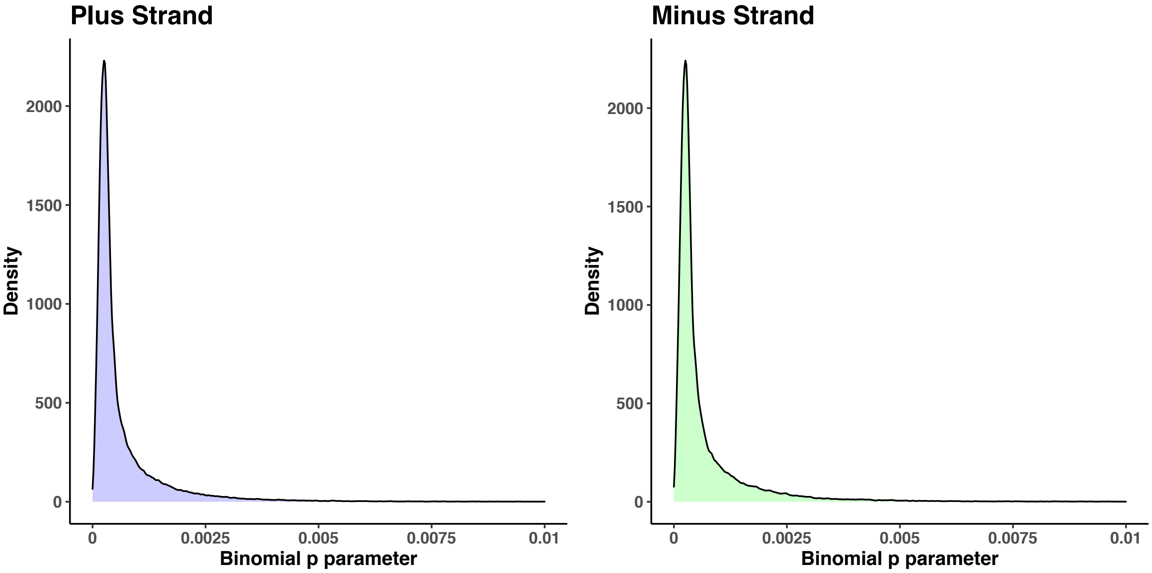

Figure S4. Density plots illustrating the distribution of the binomial p parameter used to model error rates across the entire target panel. The binomial p parameters shown here were calculated using 16 cord blood samples as controls. For every modeled allele, p was defined as the sum of alternative bases divided by the sum of reference bases across the controls.

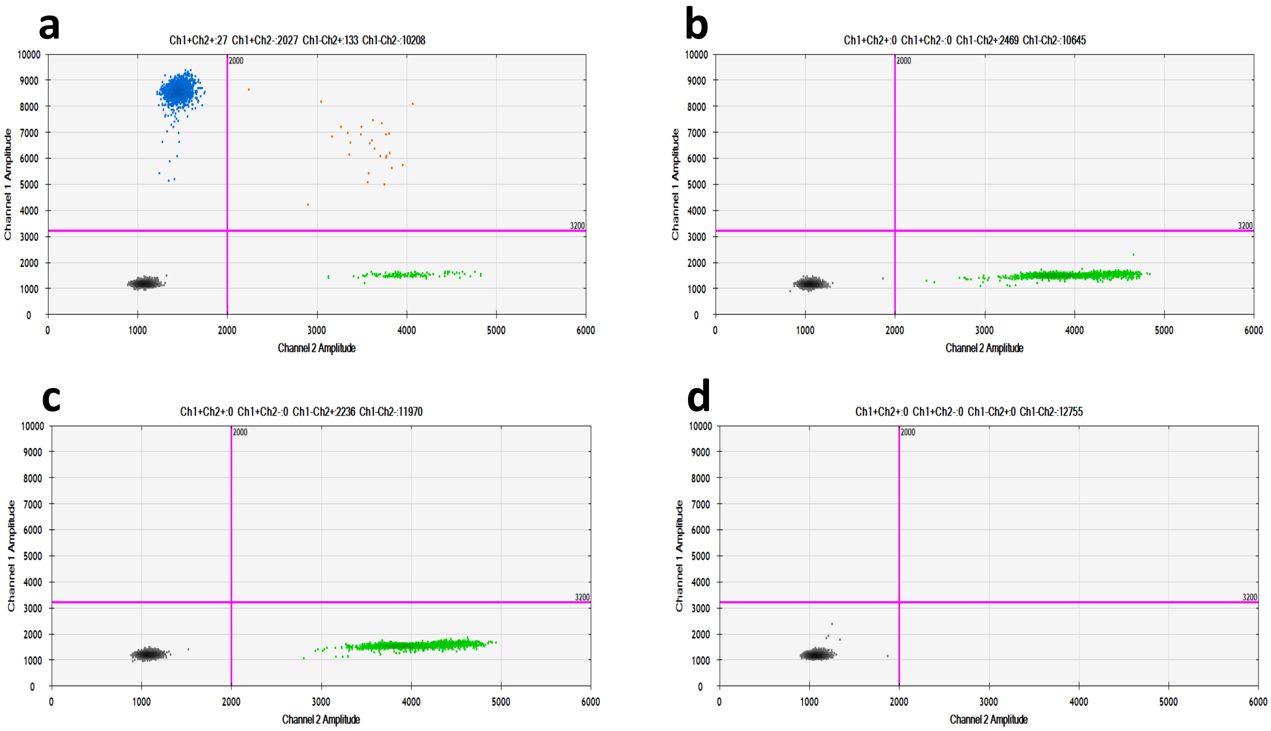

Figure S5. ddPCR results for the oncogenic false-positive variant JAK2V617F, flagged as “Potential index-hopping". **a** Sample SE16-015 served as a positive control. **b** Inability to detect the mutated allele from the test sample, SE16-0218, where the mutation was flagged as an error that likely arose due to sample-index misassignment. **c** Cord blood DNA was used as a negative control. **d** A water sample served as “blank”. Blue dots correspond to the FAM fluorophores that marks the detection of the mutated allele. Green dots correspond to the HEX fluorophores that marks the detection of the wild type allele.

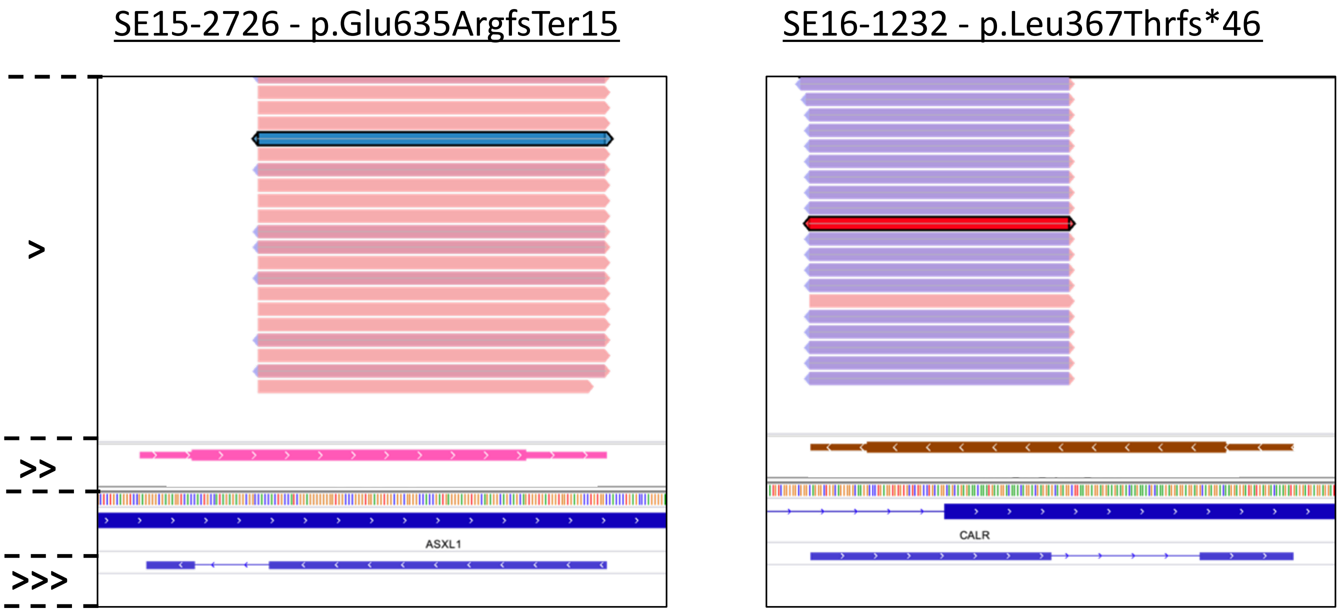

Figure S6. Misidentification of variants due to analytical artifacts. Two examples illustrate false negative calls that originated due to incorrect alignment of reads to the reference genome. > indicates alignment of the reads to the reference genome using BWA-mem. Of note, the reads for these specific genomic loci were extracted from the alignment file using SmMIP-tools' supplemental code for visual inspection. >> indicates illustration of smMIPs targeting ASXL1 and CALR. >>> indicates the correct alignment of the reads to the reference genome as determined by the BLAT alignment algorithm. BLAT alignment results are shown for those reads that are colored in blue and red for ASXL1 and CALR, respectively.

Feature	smMIP-tools	Mipgen[1]	BWA[2]	FastQC[3]	FastProNGS[4]	SAMtools[5]	GATK HaplotypeCaller[6]	Vardict[7]	iDES[8]	Pindel[9]	UMI-tools[10]	Annovar[11]	Integrative Genomics Viewer[12]	Strelka2[13]	hemoMIPs[14] pipeline
**Purposely developed for smMIP-derived data**	Yes	Yes	No	No	No	No	No	No	No	No	No	No	No	No	Partially, as it relies on existing tools that were not designed specifically for smMIP-derived data
**Probe design**	No	Yes	No	No	No	No	No	No	No	No	No	No	No	No	No
**Read alignment to the genome**	No, relies on external tools such as BWA	Aligns sequences to the genome as part of the smMIP design process	Yes	No	No	No	No	Relies on exteral tools such as BWA. Excute local realignments to improve variant detection	No	Relies on exteral tools such as BWA. Excute local realignments to improve variant detection	No	No	No	Relies on exteral tools such as BWA. Excute local realignments to improve variant detection	Yes, using BWA-mem
**Data quality assessment and reporting :**															
1) Sample level	Yes	Yes	No	Yes	Yes	No	No	Yes	Yes	No	No	No	No	Yes	Yes
2) Probe level	Yes	Yes	No	No	No	No	No	No	No	No	No	No	No	No	Yes
3) Sequencing batch level	Yes	No	No	No	No	No	No	No	No	No	No	No	No	No	No
**Read preprocessing and filtering :**															
1) Chimeric reads	Yes	No	No	No	No	No	No	No	No	No	No	No	No	No	No
2) Self-annealing probes	Yes	No	No	No	Possible with the correct user	No	No	No	No	No	No	No	No	No	No
3) Other, not smMIP specific (e.g. quality and alignment flag filters)	Yes	Supplementary code is provided to generate SAM files detailing, off-target, soft-cliped, discordant_arms, unpaired, improper_pair read, etc… (Refered in Mipgen's Github page)	No	No	Yes	Yes	Yes	Yes	No	No	No	No	No	Yes	Yes
4) Clean SAM/BAM output*	Yes	Yes	No	No	Yes	Yes	No	No	No	No	Yes			No	Yes with MIP-arm sequence trimmed
**Base call summaries (e.g. pileup) output***	Yes	No	No	No	No	Yes	No	No	Yes	No	No	No	No	No	No
**Variant calling :**															
1) Germline mutations	Yes. Might deliver sub-optimal results for highly common germline mutations if matched normal mode is not used	No	No	No	No	No	Yes	Yes	No	Yes	No	No	No	Yes	Yes, using GATK
2) Somatic SNVs	Yes	No	No	No	No	No	No	Yes	Yes	No	No	No	No	Yes	Yes, using GATK
3) Somatic Indels	Yes	No	No	No	No	No	No	Yes	No	Yes	No	No	No	Yes	Yes, using GATK
4) Structural variants	No	No	No	No	No	No	No	Yes	No	Yes	No	No	No	Yes	Coverage analysis, using a designated reference sequence file describing only the targeted structural variants
**Probabilistic approach to analyze error profiles for rare event detection**	Yes. Using a single control sample, a cohort of controls or the experimental cohort itself	No	No	No	No	No	No	Yes, tumor-normal calling mode	Yes. Designed to work with a cohort of controls	Yes, can compare tumor to normal	No	No	No	Yes, tumor-normal calling mode	No
**Use of prior knowledge concerning recurrent cancer mutations to improve their detection**	Yes	No	No	No	No	No	No	No	No	No	No	No	No	No	No
**Flagging system for variant ranking and prioritization**	Yes	No	No	No	No	No	Yes	Yes	No	Minimal **	No	No	No	Yes	Yes
**Unique molecular identifiers (UMI) :**															
1) Identify reads with compromised UMI sequences	Yes	No	Supplementary code is provided to form tag-defined read groups	No	No	No	No	No	No	No	Yes	No	No	No	No
2) Construction of consensus sequences	Yes	Supplementary code is provided to form tag-defined read groups	No	No	No	No	No	By genomic location only. Not by UMIs	No	No	Yes	No	No	No	Based on overlap of paired-end reads. Not by UMIs
3) Identify errors due to sample index misassignment	Yes	No	No	No	No	No	No	No	No	No	No	No	No	No	No
**Variant annotation**	Comprehensive annotation for SNVs. Genomic location annotation for indels	No	No	No	No	No	Can integrate different annotations if were generated externally	Variant type (e.g. SNVs, Insertion)	No	Variant type (e.g. SNVs, Insertion)	Variant type (e.g.inversion, or tandem duplication)	Yes	No	Variant type (e.g. SNVs, Insertion)	Reports annotations concerning pathogenicity and existence of common variants
**Sequences visualization for review**	No	No	No	No	No	No	No	No	No	Yes	No	No	Yes	No	No

* To be used with other tools for subsequent analysis if those better fit user's requirements

** Include information concerning the appearance of variants in other samples if multiple samples were tested. This information can potentially be used to identify recurrent errors

Software references

1 Boyle, E. A., O'Roak, B. J., Martin, B. K., Kumar, A. & Shendure, J. MIPgen: optimized modeling and design of molecular inversion probes for targeted resequencing. *Bioinformatics* **30**, 2670–2672 (2014).

2 Li, H. & Durbin, R. Fast and accurate long-read alignment with Burrows-Wheeler transform. *Bioinformatics* (2010). doi:10.1093/bioinformatics/btp698

3 Babraham Bioinformatics. FastQC:A quality control tool for high throughput sequence data. Available at: http://www.bioinformatics.babraham.ac.uk/projects/fastqc/. .

4 Liu, X. *et al.* FastProNGS: fast preprocessing of next-generation sequencing reads. *BMC Bioinformatics* **20**, 345 (2019).

5 Li, H. *et al.* The Sequence Alignment/Map format and SAMtools. *Bioinformatics* (2009). doi:10.1093/bioinformatics/btp352

6 Poplin, R. *et al.* Scaling accurate genetic variant discovery to tens of thousands of samples. *bioRxiv* 201178 (2018). doi:10.1101/201178

7 Lai, Z. *et al.* VarDict: a novel and versatile variant caller for next-generation sequencing in cancer research. *Nucleic Acids Res.* **44**, e108 (2016).

8 Newman, A. M. *et al.* Integrated digital error suppression for improved detection of circulating tumor DNA. *Nat. Biotechnol.* (2016). doi:10.1038/nbt.3520

9 Ye, K., Schulz, M. H., Long, Q., Apweiler, R. & Ning, Z. Pindel: a pattern growth approach to detect break points of large deletions and medium sized insertions from paired-end short reads. *Bioinformatics* **25**, 2865–2871 (2009).

10 Smith, T., Heger, A. & Sudbery, I. UMI-tools: modeling sequencing errors in Unique Molecular Identifiers to improve quantification accuracy. *Genome Res.* **27**, 491–499 (2017).

11 Wang, K., Li, M. & Hakonarson, H. ANNOVAR: functional annotation of genetic variants from high-throughput sequencing data. *Nucleic Acids Res.* **38**, e164–e164 (2010).

12 Robinson, J. T. *et al.* Integrative genomics viewer. *Nat. Biotechnol.* **29**, 24–26 (2011).

13 Kim, S., Scheffler, K., Halpern, A.L. et al. Strelka2: fast and accurate calling of germline and somatic variants. *Nat Methods* **15**, 591–594 (2018).

14 Kleinert P, Martin B, Kircher M. HemoMIPs-Automated analysis and result reporting pipeline for targeted sequencing data. *PLoS Comput Biol.* 2020 Jun 4;16(6):e1007956. doi: 10.1371/journal.pcbi.1007956.
